# Binocular Summation in Healthy Presbyopic Adults: Contrast Sensitivity and Defocus Curves

**DOI:** 10.3390/jpm16090465

**Published:** 2026-09-09

**Authors:** Vanesa García-del-Castillo, Mónica Alamo-Peña, César Albarrán-Diego, Cecilia Arnaiz-Schmitz, Nuria Garzón, María García-Montero

**Affiliations:** 1 Department of Optometry and Vision, Complutense University of Madrid, 28037 Madrid, Spain; vagarc03@ucm.es (V.G.-d.-C.); mondelal@ucm.es (M.A.-P.); mgarc01@ucm.es (M.G.-M.); 2 Department of Optics and Optometry and Vision Sciences, University of Valencia, 46100 Burjassot, Spain; cesar.albarran@uv.es; 3 Department of Biodiversity, Ecology and Evolution, Complutense University of Madrid, 28037 Madrid, Spain; caschmitz@ucm.es

**Keywords:** binocular summation, contrast sensitivity, defocus curve

## Abstract

**Background/Objective:** Binocular summation, the improvement in visual performance under binocular compared with monocular viewing, depends on the visual task and the degree of optical degradation of the retinal image. Its behavior across the full defocus curve in presbyopia remains poorly characterized. The purpose is to quantify binocular summation in healthy presbyopic adults by assessing contrast sensitivity (CS) at intermediate and near distances and by comparing monocular and binocular defocus curves to evaluate the effect of binocularity on depth of focus, the area under the curve (AUC), and the binocular summation ratio (BSR). **Methods:** Thirty healthy presbyopic participants (mean age 53.7 ± 5.7 years, range 45–64) were included in this observational, cross-sectional study. CS was measured with the Pelli–Robson chart at 1 m and 40 cm, and monocular and binocular defocus curves were obtained from −3.50 D to +1.50 D in 0.50 D steps. BSR, AUC and depth of focus at the 0.2 and 0.1 logMAR thresholds were calculated. Monocular and binocular values were compared with paired *t*-tests, applying a Bonferroni correction. **Results:** Binocular CS was significantly higher than monocular CS at both distances (adjusted *p* < 0.025), with a large, distance-independent BSR (1.68 at 1 m, 1.69 at 40 cm). BSR exceeded unity at every defocus level (10–21% gain), but after correction the binocular advantage in visual acuity remained significant only within the central range (−0.50 D to +1.00 D). At the participant level, binocularity increased the AUC at the 0.1 logMAR threshold (+50.5%, *p* = 0.037, d = 0.40), whereas the increase at the 0.2 logMAR threshold (+52.2%) did not reach statistical significance (*p* = 0.107). **Conclusions:** Binocularity consistently enhances visual performance in presbyopic adults, for contrast sensitivity and, with a pattern of significant improvement concentrated in the central defocus range, for visual acuity, supporting a more personalized evaluation of presbyopia.

## 1. Introduction

Binocular summation refers to the improvement in visual performance obtained under binocular viewing compared with monocular viewing [1,2], and arises from the cortical integration of signals presented independently to each eye [3]. This binocular advantage is conventionally quantified by the binocular summation ratio (BSR), defined as the ratio of binocular to monocular visual performance [1,4,5]. A BSR greater than unity indicates binocular summation, whereas values below unity indicate binocular inhibition, that is, poorer binocular than monocular performance for a given visual outcome [4,6].

The magnitude of binocular summation depends on the visual function being evaluated. A quadratic summation model was proposed by Legge (1984) as a fundamental theoretical rule describing binocular contrast summation, and under the assumption that both eyes have equal monocular sensitivity, the model states that the monocular contrast signals combine quadratically to form an effective binocular contrast [7]. For near-threshold contrast detection tasks, the model predicts a theoretical binocular summation ratio of √2 (≈1.41), a prediction that Legge experimentally confirmed using sinusoidal grating stimuli [8].

For contrast sensitivity (CS), a near threshold detection task, binocular summation generally approaches the theoretical prediction of the quadratic summation model, whereas for high-contrast visual acuity (VA), a suprathreshold resolution task, the binocular gain is considerably smaller [1,4,5,8].

From a clinical standpoint, defocus curves (DCs) have become the gold standard tool for evaluating visual performance across a range of simulated distances, particularly in the assessment of presbyopia correction strategies such as multifocal or extended depth of focus (EDOF) intraocular lenses (IOLs), and age-related changes in visual function [9,10,11]. A better understanding of binocular summation across different levels of defocus may support a more personalized evaluation of functional vision and help optimise patient-specific presbyopia correction strategies.

The DCs are typically obtained under monocular conditions. However, their binocular counterpart provides more ecologically valid information, since daily visual tasks are inherently binocular. Despite this, normative binocular defocus curve data in presbyopic phakic participants remain scarce. To our knowledge, only one study has previously reported binocular defocus curves in a presbyopic cohort, and it was restricted to participants with emerging presbyopia up to 48 years of age [10].

Furthermore, while binocular summation of CS has been well characterized in adult populations across a broad age range [4,5], the behavior of binocular summation across the full defocus curve, encompassing distances from near to far, has not been described in detail. The relationship between defocus-induced blur and the ability of the binocular visual system to integrate degraded monocular inputs in presbyopia represents a clinically relevant but understudied phenomenon [12].

Age-related changes affect both the shape of defocus curves and the area under the curve (AUC), with depth of focus progressively decreasing from one age decade to the next [9]. Contrast sensitivity is similarly affected by ageing, yet the binocular cortical integration mechanisms appear to remain functionally preserved even in older adults [4]. In line with this, binocular viewing is almost always superior to monocular viewing, and this advantage is most apparent under low-contrast conditions, where signal amplification is most beneficial [13].

Within the personalized medicine framework, presbyopia correction has progressively shifted from generic, population-averaged recommendations toward individualized strategies that account for the specific visual profile of each patient, including the selection and power calculation of multifocal or EDOF intraocular lens platforms, near addition power, and monovision or blended vision approaches. However, the clinical protocols and diagnostic criteria supporting these individualized decisions still rely predominantly on monocular defocus curves, despite binocular viewing being the physiological condition under which patients experience functional vision in daily life. Quantifying how binocular summation of contrast sensitivity and visual acuity behaves across the full defocus range therefore provides objective, patient-specific parameters, namely the binocular summation ratio and the binocular area under the curve, that can be incorporated into presurgical and optometric assessment protocols, moving presbyopia management beyond monocular, population-based criteria toward a genuinely individualized approach.

The present study aimed to quantify binocular summation in healthy presbyopic participants by assessing CS at intermediate and near viewing distances and by comparing monocular and binocular defocus curves to evaluate the effect of binocularity on depth of focus, the area under the curve, and the binocular summation ratio across the full range of defocus.

## 2. Materials and Methods

### 2.1. Study Design and Participants

An observational, cross-sectional, within-subject comparative study was conducted in healthy presbyopic participants. Data collection was conducted between September 2025 and March 2026. The study was approved by the Ethics Committee of Hospital Clínico San Carlos, Madrid, Spain (Internal code: 23/040-E), and all procedures conformed to the tenets of the Declaration of Helsinki. Written informed consent was obtained from all participants prior to enrolment.

Participants were recruited at the Faculty of Optics and Optometry, Universidad Complutense de Madrid. Inclusion criteria were: age 45–65 years, presbyopic status, and monocular distance corrected monocular VA (mCDVA) 0.00 logMAR or better in both eyes. Exclusion criteria included any ocular or systemic condition affecting visual performance, history of amblyopia, strabismus, or previous ocular surgery.

### 2.2. Visual Assessment Procedure

A complete refractive workup was performed for all participants by a single examiner and all examinations were performed following the same standardized protocol. Objective refraction was obtained with the Topcon KR-7000P autorefractor (Topcon Corp., Tokyo, Japan) under photopic conditions (85 cd/m^2^). Subjective refraction was performed using a phoropter and an ETDRS chart (Precision Vision, Woodstock, IL, USA) at 4 m (with +0.25 D compensation for the finite working distance), employing the fogging technique and Jackson crossed cylinders (JCCs) to achieve maximum plus to maximum VA (MPMVA). Near addition was determined with fused crossed cylinders (FCCs) at 40 cm under photopic conditions. All subsequent visual assessments were performed using the optimal subjective refractive correction for the corresponding testing distance. Testing was performed under the same photopic illumination throughout the experimental session.

Sensory ocular dominance at distance and near was established by alternately interposing a +1.00 D lens over each eye while the participant fixated on an ETDRS letter using distance and near ETDRS charts at 4 m and 40 cm, respectively. The eye experiencing greater blurring discomfort with the lens interposed was designated the dominant eye. For participants without a clear sensory preference, motor ocular dominance was determined using the hole-in-card test.

### 2.3. Contrast Sensitivity

CS was measured with the Pelli–Robson Contrast Sensitivity Chart under photopic conditions at two distances. Two calibrated versions manufactured by Precision Vision (Woodstock, IL, USA) were used: the standard 1 m chart and the near 40 cm chart. At 1 m, participants wore their full distance subjective correction with an additional +0.75 D lens to compensate for the accommodative demand at this testing distance, following the manufacturer’s recommendations. At 40 cm, the near-calibrated chart was used with the full near correction (distance correction plus the subjective near addition). Monocular CS was assessed using only the task-appropriate dominant eye at each distance—the distance-dominant eye at 1 m and the near-dominant eye at 40 cm—followed by binocular assessment at each distance. The non-dominant eye was not tested monocularly for CS at either distance, as this was not part of the original protocol. Scoring followed the standard Pelli–Robson triplet rule: a specific logarithmic value is assigned to each correctly read triplet, with credit given when at least two of the three letters in a triplet are correctly identified, following the manufacturer’s official scoring template.

Only one Pelli–Robson chart version was available at 1 m; at 40 cm, multiple chart versions were available. Ambient room illumination was held constant at approximately 200–250 lux throughout testing. During monocular testing, the fellow eye was occluded with an opaque occluder lens mounted in a trial frame. Monocular testing was always performed before binocular testing. A brief rest interval separated the two conditions, with all measurements completed within a single session. The order of CS and defocus curve assessment was randomized across participants. The examiner was aware of the viewing condition (monocular/binocular) being tested at each step; the study was not examiner-masked.

#### BSR for Contrast Sensitivity

BSR_CS_ was calculated using the task-appropriate dominant-eye monocular CS value as the reference (distance-dominant eye at 1 m, near-dominant eye at 40 cm), with all values converted from logCS to linear scale:

BSR_CS_ = 10^(log CS_binocular_ − log CS_monocular_)

### 2.4. Defocus Curves

The defocus curve was assessed at 4 m, adding a + 0.25 D lens to compensate for the testing distance. Before testing DC, DCVA was determined through subjective refraction using the MPMVA method with the Early Treatment Diabetic Retinopathy Study (ETDRS) chart (Precision Vision, USA) at 4 m. Spherical lenses ranging from −3.50 D to +1.50 D in 0.50 D steps were superimposed on the distance subjective refraction. VA at each defocus level was recorded in logMAR notation using the letter-by-letter scoring method (0.02 logMAR per letter). Three different ETDRS chart versions were used to minimize learning effects to assess the monocular (using the distance-dominant eye) and the binocular curves, since randomizing letter sequences and/or lens presentation order has been shown to be necessary to prevent memory-related overestimation of visual performance in defocus-curve testing [14]. For the monocular defocus curve, the distance-dominant eye was evaluated across all 11 defocus levels; this was the same eye used for CS testing at 1 m. As with CS testing, monocular defocus curves were always assessed before binocular curves, with a brief rest interval between conditions, all within the same session; the examiner was not masked to viewing condition.

#### 2.4.1. Calculation of BSR for Visual Acuity

To maintain consistency with the BSR_CS_ calculation convention (BSR > 1 = binocular advantage), VA logMAR values were converted to the linear minimum angle of resolution (MAR = 10^(logMAR)) and BSR_VA_ was computed for each defocus, as:

BSR_VA_ = MAR_monocular_/MAR_binocular_

This inversion ensures that a BSR > 1 reflects binocular superiority despite the inverse scoring direction of the logMAR scale.

#### 2.4.2. Area Under the Curve and Depth of Focus

The area under the defocus curve was used as a summary measure of overall visual performance across the range of defocus tested. AUC was calculated in RStudio (R version 4.5.0) using the trapezoidal rule for two predefined visual acuity thresholds: 0.2 logMAR, representing the limit of functional vision, and 0.1 logMAR, representing optimal visual performance. The 0.2 logMAR (20/32 Snellen) threshold corresponds to the functional-vision limit adopted in the AAO consensus for depth-of-focus assessment [11], while relative criteria of this type are well established for quantifying depth of focus from defocus curves [14].

For descriptive purposes, AUC and depth of focus were also derived from the group-mean monocular and binocular defocus curves to facilitate comparison with previous studies using this approach. Depth of focus was defined as the continuous range of defocus over which VA remained better than each predefined threshold (0.2 and 0.1 logMAR). The exact defocus values at which each threshold was reached, and thus the corresponding cut-off points, were determined by linear interpolation between adjacent defocus levels.

For the individual-level analysis, AUC was calculated separately for each participant and viewing condition using the trapezoidal rule for the same two predefined VA thresholds. Only portions of the defocus curve with VA better than the selected threshold were included in the calculation. AUC was computed as the integral, over the defocus axis (D), of the vertical distance between the selected VA threshold and the defocus curve, restricted to the region where VA was better than the threshold: AUC = ∫(threshold − VA(defocus)) d(defocus), evaluated only where VA(defocus) < threshold. The resulting individual AUC values are summarized as mean ± SD and were used for paired monocular–binocular statistical comparisons.

Consequently, AUC is expressed in logMAR·D, and larger AUC values indicate a better visual performance over a broader range of defocus, in line with the direction of BSR_CS_ and BSR_VA_ reported elsewhere in this manuscript.

### 2.5. Statistical Analysis

Due to the exploratory nature of this pilot study, a formal sample size calculation was not performed a priori. The sample size is deemed adequate to provide preliminary estimates to guide the design and planning of future confirmatory studies. A target sample size of 30 participants was established based on feasibility and was considered sufficient to provide preliminary estimates of binocular summation and variability for the planning of future confirmatory studies.

Descriptive data are reported as mean ± SD. Normality was assessed on the paired differences, not on raw values, using the Shapiro–Wilk test; where differences departed from normality, Wilcoxon signed-rank tests were performed as a sensitivity analysis alongside paired Student’s *t*-tests. Monocular versus binocular VA across the defocus curve was analyzed with a two-way repeated-measures ANOVA (condition × defocus level), with Greenhouse–Geisser (GG) correction where sphericity was violated, followed by Bonferroni-corrected planned pairwise contrasts at each defocus level, reported with 95% confidence intervals and Cohen’s d. Effect sizes for the repeated-measures ANOVA are reported as generalized eta squared (generalized η^2^), which incorporates between-subject variance and is recommended for repeated-measures designs to allow comparability with between-subjects studies. Monocular versus binocular CS was compared at each distance using paired *t*-tests and Wilcoxon tests. BSR for CS was compared between the two testing distances using a paired *t*-test and Wilcoxon test. AUC was calculated per participant, not from the group-mean curve, at both VA thresholds and compared between conditions using paired *t*-tests, Wilcoxon tests, and bootstrap 95% confidence intervals for group means. Cut-off points and depth of focus derived from the group-mean monocular and binocular defocus curves are reported descriptively and were not subjected to inferential statistical comparison. Bootstrap 95% confidence intervals for the group-mean cut-off points were also calculated. Individual depth-of-focus comparisons were not performed because, in some participants, one of the predefined VA thresholds was not reached within the tested defocus range (−3.50 to +1.50 D), precluding determination of both cut-off points by interpolation.

The nominal level of statistical significance was set at α = 0.05. To control the family-wise error rate, Bonferroni corrections were applied separately to each family of comparisons. For the 11 monocular–binocular comparisons performed across the defocus curve, the corrected significance threshold was α = 0.05/11 = 0.0045. The two monocular–binocular CS comparisons, one at each testing distance, constituted a separate family, resulting in a corrected significance threshold of α = 0.025.

Statistical analyses were performed using Statgraphics Centurion 19 (Statgraphics Technologies, Inc., The Plains, VA, USA). Defocus curves and AUC plots were generated using R in RStudio (R version 4.5.0). Ratio calculations were performed using Microsoft Excel (Office 2024).

## 3. Results

### 3.1. Participants

The sample comprised 30 participants (16 women, 53.3%; 14 men, 46.7%) with a mean age of 53.73 ± 5.71 years (range: 45–64). Descriptive data are summarized in Table 1.

### 3.2. Contrast Sensitivity

Binocular CS was significantly higher than monocular CS at both distances (1 m: t = 6.07, *p* = 1.3 × 10^−6^, Wilcoxon *p* = 6.8 × 10^−5^, d = 1.11; 40 cm: t = 10.78, *p* = 1.2 × 10^−11^, Wilcoxon *p* = 2.9 × 10^−6^, d = 1.97). Paired differences were not normally distributed at either distance (Shapiro–Wilk *p* < 0.005 both), supporting the Wilcoxon results as a robustness check. Monocular CS was 1.755 ± 0.168 logCS at 1 m and 1.695 ± 0.137 logCS at 40 cm; binocular CS was 1.945 ± 0.062 logCS and 1.910 ± 0.096 logCS, respectively. The mean binocular advantage was 0.190 logCS at 1 m (95% CI 0.126–0.254) and 0.215 logCS at 40 cm (95% CI 0.174–0.256). Full BSR_CS_ data are presented in Table 2. BSR_CS_ did not differ significantly between testing distances (t = −0.11, *p* = 0.915; Wilcoxon *p* = 0.829).

### 3.3. Defocus Curves

**Figure 1:**
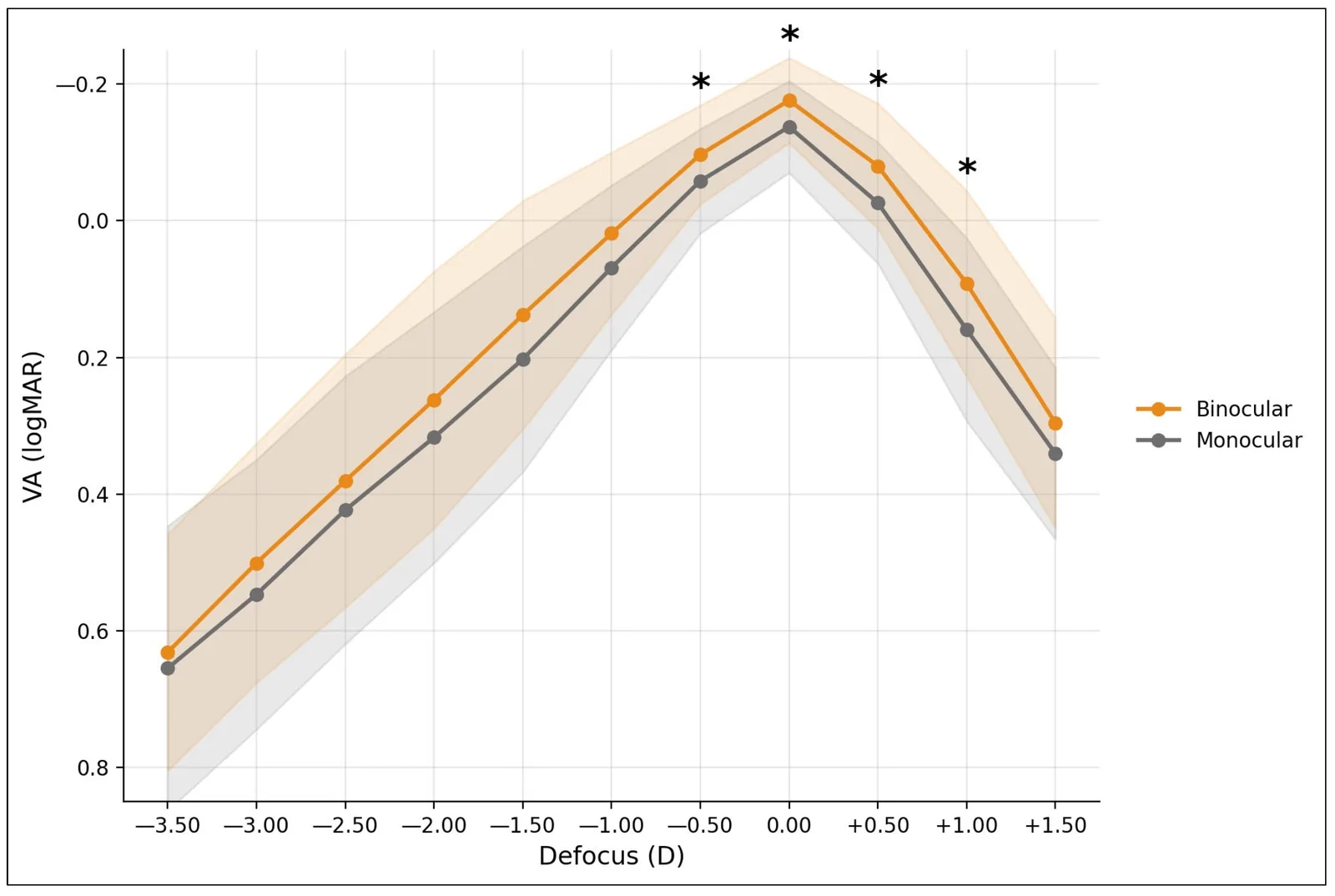
Monocular and binocular defocus curves. Points represent mean visual acuity; shaded bands represent ±1 SD. Binocular visual acuity showed lower logMAR values than monocular visual acuity across the defocus range. Asterisks indicate statistically significant paired monocular–binocular differences based on the Bonferroni-corrected significance threshold (*p* < 0.0045; 11 comparisons).

Both monocular and binocular defocus curves peaked at zero defocus (Figure 1). At this level, binocular VA (−0.18 ± 0.06 logMAR) exceeded monocular VA (−0.14 ± 0.07 logMAR). The binocular curve was consistently superior to the monocular curve across the entire defocus range tested. Table 3 presents full comparative data.

A two-way repeated-measures ANOVA (condition × defocus level) showed significant main effects of condition (F(1,29) = 17.44, *p* < 0.001, generalized η^2^ = 0.026) and defocus level (F(10,290) = 201.09, Greenhouse–Geisser corrected *p* < 0.001, generalized η^2^ = 0.741), but no significant condition × defocus interaction (F(10,290) = 0.55, GG-corrected *p* = 0.673, generalized η^2^ = 0.002), indicating that the magnitude of the binocular advantage did not differ significantly across the defocus range. Sphericity was significantly violated (Mauchly’s χ^2^ = 332.3, *p* < 0.001), justifying the Greenhouse–Geisser correction.

After Bonferroni correction for multiple comparisons (α = 0.0045), binocular visual acuity was significantly better than monocular visual acuity at −0.50, 0.00, +0.50, and +1.00 D. Differences at the remaining defocus levels did not reach statistical significance. These planned contrasts are reported with 95% CIs, Cohen’s d, and Wilcoxon sensitivity analyses in Table 3, given non-normal paired differences at several defocus levels.

#### 3.3.1. BSR Across the Defocus Curve

Mean BSR_VA_ values exceeded 1.0 at all defocus levels, indicating binocular summation on average throughout the defocus curve (Table 4). The lowest mean BSR_VA_ values were observed at −0.50 and 0.00 D (1.10 ± 0.16 and 1.10 ± 0.09, respectively), corresponding to an average binocular gain of approximately 10% and the lowest intersubject variability. The numerically highest mean BSR_VA_ was observed at −1.50 D (1.21 ± 0.38), corresponding to a 21% binocular gain. Away from the central defocus range, BSR_VA_ variability generally tended to be greater, reaching an SD of 0.45 at −3.50 D. It should be noted that the statistical significance of the binocular advantage is reported in terms of the monocular versus binocular VA comparison rather than by testing BSR_VA_ values against unity.

#### 3.3.2. Area Under the Curve and Depth of Focus

**Figure 2:**
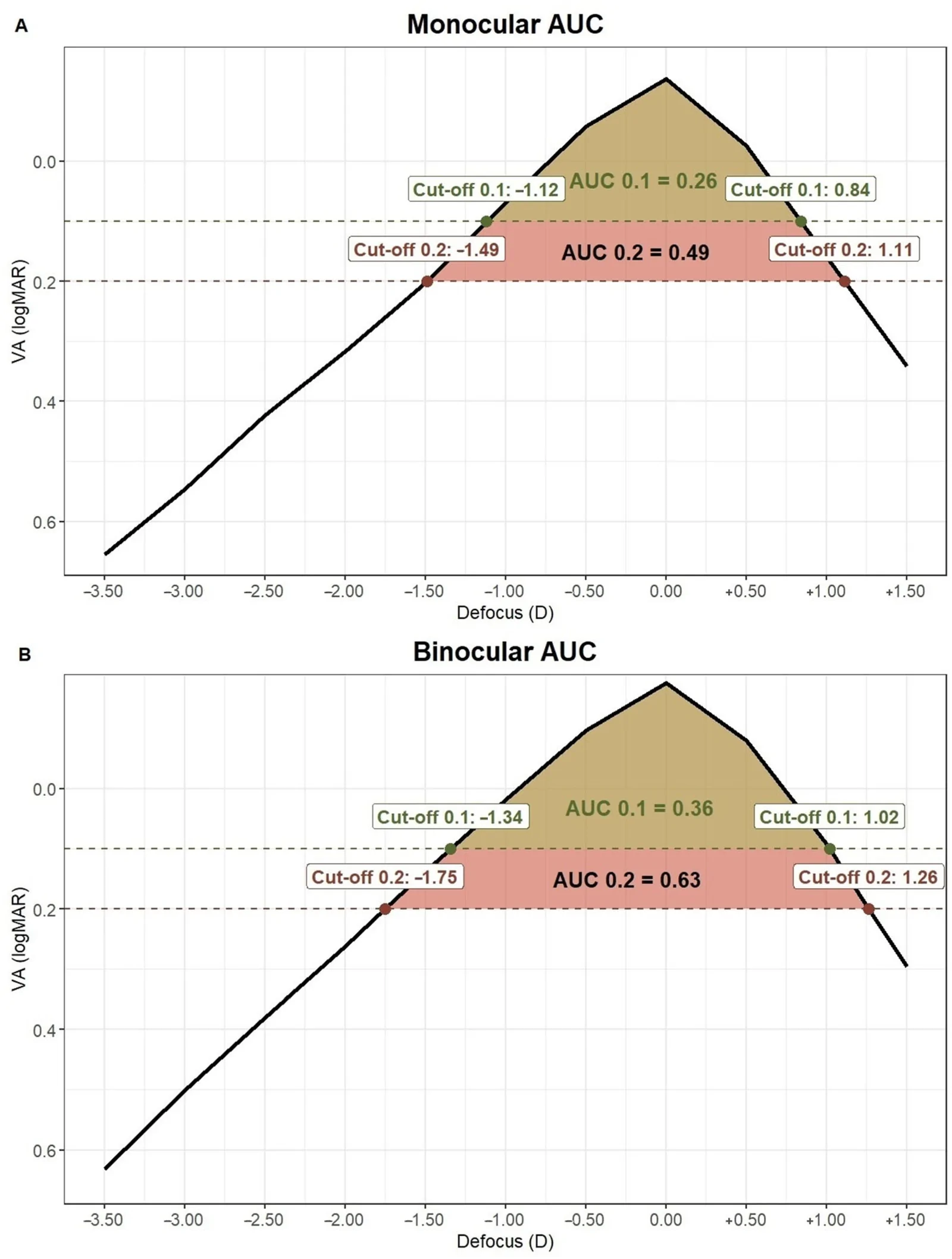
Monocular (**A**) and binocular (**B**) areas under the curve (AUC) for two visual acuity (VA) reference thresholds: ≤0.1 logMAR (green) and ≤0.2 logMAR (red). AUC values indicate the magnitude of the useful vision area. Cut-off points indicate the defocus level (D) at which the curve intersects each VA reference line.

AUC was calculated per participant rather than from the group-mean curve, to allow paired statistical comparison. At the 0.1 logMAR threshold, binocular AUC (0.274 ± 0.238) was significantly greater than monocular AUC (0.182 ± 0.198; t = 2.19, *p* = 0.037, Wilcoxon *p* = 0.013, d = 0.40, mean difference 0.092, 95% CI 0.006–0.177). At the 0.2 logMAR threshold, binocular AUC (0.458 ± 0.403) was numerically greater than monocular AUC (0.301 ± 0.309), but the difference did not reach statistical significance (t = 1.67, *p* = 0.107, Wilcoxon *p* = 0.075, d = 0.30, mean difference 0.157, 95% CI −0.036–0.349) and should be interpreted as a non-significant trend given the modest sample size. For descriptive purposes, AUC was also calculated directly from the group-mean defocus curves, as shown in Figure 2. At the 0.1 logMAR threshold, the group-mean curve AUC was 0.26 logMAR·D monocularly and 0.36 logMAR·D binocularly. At the 0.2 logMAR threshold, the corresponding values were 0.49 and 0.63 logMAR·D, respectively. At both thresholds, cut-off points were displaced toward greater negative defocus under binocular viewing. At the 0.1 logMAR threshold, monocular and binocular curves crossed at −1.12 D (95% CI −1.31 to −0.96)/+0.84 D (95% CI 0.75 to 0.96) and −1.34 D (95% CI −1.59 to −1.15)/+1.02 D (95% CI 0.90 to 1.14), respectively (depth of focus 1.96 D and 2.36 D). At the 0.2 logMAR threshold, the corresponding values were −1.49 D (95% CI −1.76 to −1.30)/+1.11 D (95% CI 0.98 to 1.23) monocularly and −1.75 D (95% CI −2.02 to −1.52)/+1.27 D (95% CI 1.15 to 1.39) binocularly (depth of focus 2.60 D and 3.01 D).

## 4. Discussion

This study characterizes the binocular summation in healthy presbyopic phakic subjects aged 45–65 years by assessing two complementary aspects of visual function: contrast sensitivity and visual acuity across the defocus curve. The findings confirm that binocular viewing provides a consistent functional advantage over monocular viewing. However, the magnitude of this advantage differed according to the visual function assessed, being greatest for contrast sensitivity and more modest for high contrast visual acuity measured across increasing levels of defocus. These findings are consistent with the concept that binocular summation depends on the characteristics of the visual task and the degree of optical degradation of the retinal image.

For CS, our BSR values (1.68 at 1 m and 1.69 at 40 cm) exceed the 40–51% binocular improvement typically described for normal adults, well beyond the theoretical quadratic prediction of √2 (41%) [1,4,5]. Xiong et al. [5] demonstrated that this robust summation rule applies uniformly to normal vision and to a variety of common eye diseases, suggesting that the cortical mechanism of contrast summation is functionally preserved even when monocular sensory input is degraded. It should be noted, however, that some studies have reported an age-related reduction in binocular summation when younger and older adults are directly compared [15]. In our cohort, the large and distance-independent BSR_CS_ suggests that, within the 45–65 year range studied, this contrast-summation mechanism remains largely effective. BSR_CS_ did not differ significantly between 1 m and 40 cm (t = −0.11, *p* = 0.915; Wilcoxon *p* = 0.829), suggesting similar binocular contrast summation at the two testing distances. In contrast, the repeated-measures analysis of the VA defocus curve showed no significant condition × defocus interaction, indicating that the magnitude of binocular advantage in VA did not differ significantly across the defocus range, even though only selected defocus levels reached statistical significance in the Bonferroni-corrected pairwise comparisons.

Regarding defocus curve analysis, the binocular superiority in VA at zero defocus (approximately 0.04 logMAR) is consistent with prior reports from Shafer et al. [10], who found comparable binocular VA values (−0.16 ± 0.06 logMAR) in a slightly younger presbyopic cohort (46–48 years). The agreement between both studies reinforces the observation by Baoud et al. [9] that performance at the focal peak remains relatively preserved across the studied age range under optimal correction. This is congruent with population data in healthy eyes showing that best-corrected VA remains essentially stable until the mid-fifties, with the first significant age-related break-point appearing only at 55–59 years [16], and with the wider notion that normal VA in healthy eyes exceeds the conventional 6/6 standard [17].

At all tested defocus levels between −0.50 and +1.00 D, binocular VA was significantly better than monocular VA based on the Bonferroni-corrected significance threshold (all *p* ≤ 0.001; α = 0.0045), although the magnitude of the binocular gain was modest (10–20%; mean BSR_VA_, 1.10–1.20). The low intersubject variability suggests a highly homogeneous binocular response, consistent with the theoretical framework proposed by Frisen et al. [2] and later elaborated by Peñaloza et al. [4], who noted that high-contrast VA tasks elicit only modest binocular summation in line with early reports of a small binocular advantage for high-contrast acuity [18,19,20].

At the intermediate defocus levels of −1.00 and −1.50 D, relatively high mean BSR_VA_ values were observed, with the highest value occurring at −1.50 D (BSR_VA_ = 1.21). However, the monocular–binocular VA differences at these defocus levels did not meet the Bonferroni-corrected significance threshold (both *p* = 0.005; α = 0.0045). At these distances, presbyopia progressively degrades monocular signal quality, and the binocular cortical system may partially compensate by integrating two degraded but complementary inputs, although this trend should be interpreted with caution given the loss of statistical significance under conservative multiplicity correction. This is broadly consistent with Plainis et al. [12], who reported that binocular summation improves performance under defocus-induced blur precisely because optotypes approach the detection threshold as blur increases.

Outside the −0.50 D and +1.00 D range, statistical significance was lost after Bonferroni correction, even though BSR_VA_ values remained above unity throughout the curve (14–21%), reflecting increased intersubject variability at higher levels of defocus (SD up to ± 0.45). This pattern suggests that, at high levels of retinal blur, the quality of the monocular input to each eye becomes so degraded that cortical integration is no longer efficient for a proportion of subjects, leading to heterogeneous responses.

Participant-level AUC analysis showed a significant binocular advantage at the stricter 0.1 logMAR threshold (d = 0.40), indicating better binocular visual performance across the optimal range of defocus. At the 0.2 logMAR threshold, the numerically larger binocular advantage (+52%) did not reach statistical significance at this sample size (d = 0.30), and should be interpreted as a preliminary trend requiring confirmation in a larger cohort, rather than as a confirmed effect. For comparison with previously published defocus-curve literature, monocular and binocular AUC were also calculated from the group-mean curve, following the same approach used by Baoud et al. [9]. At the 0.2 logMAR threshold, mean-monocular AUC (0.49) was consistent with the age-related reduction reported by Baoud et al. [9], who documented AUC values ranging from 0.51 (45–50 years) to 0.33 (61–65 years); binocularity expanded this group-level AUC to 0.63 (+28.6%) and shifted the near cut-off point from −1.49 D to −1.75 D, descriptively indicating a broader range of functional visual performance. However, to allow paired statistical comparison and quantify individual variability, AUC was additionally calculated per participant. At the participant level, the binocular advantage was statistically significant at the 0.1 logMAR threshold (0.182 ± 0.198 vs. 0.274 ± 0.238; *p* = 0.037, d = 0.40) but did not reach significance at the 0.2 logMAR threshold (0.301 ± 0.309 vs. 0.458 ± 0.403; *p* = 0.107, d = 0.30), reflecting interindividual variability that is not captured when AUC is derived from the averaged curve. The AUC values derived from the group-mean curves are therefore reported for comparability with the published literature, while the participant-level analysis should be regarded as the primary basis for inferential claims in this study.

Regarding the BSR_VA_ methodology, our value at zero defocus (1.10 ± 0.09) is closely aligned with the 1.06 ± 0.01 reported by Peñaloza et al. [4] using the weighted linear summation model, supporting the established finding that both summation models yield statistically equivalent BSR estimates for VA and that BSR for VA remains relatively stable across adulthood.

Moreover, these findings confirm the functional independence of VA and CS summation mechanisms postulated by Frisen et al. [2] and supported by Peñaloza et al. [4] and Xiong et al. [5]. VA summation operates on a suprathreshold resolution task and is limited by the cortical priority for spatial precision; CS summation operates near threshold and benefits from quadratic signal amplification when interocular balance is maintained.

These findings have clinical implications for presbyopia management. Because binocular summation follows distinct patterns for VA and CS, correction strategies based solely on monocular data may not capture the functional outcome patients experience under everyday binocular viewing. The largest binocular gain, at intermediate defocus (−1.50 D), was not significant after correction but may still be relevant for customizing EDOF and multifocal IOL power profiles, near addition, and monovision or blended-vision strategies, since it marks the defocus range where binocular integration compensates most for optical degradation. Likewise, the large, distance-independent BSR_CS_, contrasting with the more modest, defocus-dependent BSR_VA_, supports incorporating both metrics into presurgical assessment to help distinguish patients likely to benefit from binocular compensation from those who may need a more symmetry-preserving correction. BSR can be obtained from brief, low-cost tests already used in optometric practice, making it feasible for preoperative counselling and follow-up. Patients with a BSR above the theoretical quadratic prediction may be better candidates for strategies that introduce interocular blur difference, such as monovision or asymmetric multifocal designs, whereas those with a lower or more variable BSR may benefit more from symmetric correction. Prospective studies combining interocular symmetry measures with BSR would help test this framework.

The present work has several limitations that should be considered when interpreting the findings. The sample size, established on the basis of feasibility for this exploratory pilot study, limits the statistical precision of subgroup comparisons. The broad age range (45–65 years) encompasses heterogeneous stages of presbyopia; a stratified analysis by decade would require substantially larger cohorts. Additionally, CS at near was assessed using the near-dominant eye, contralateral by definition to the distance-dominant eye used for CS at 1 m and for the defocus curve; this task-appropriate protocol, while clinically standard, precludes computation of an interocular ratio and limits direct comparison across outcome measures. Future designs should record monocular data for both eyes at both distances. The non-significant condition×defocus interaction and the non-significant AUC difference at the 0.2 logMAR threshold suggest the study may be underpowered to detect defocus-dependent modulation of binocular summation; a larger confirmatory sample is warranted. Factors known to modulate defocus curve shape, including pupil size and optical aberrations, were not systematically quantified, although testing conditions were standardized. Finally, the application of a conservative Bonferroni correction for the 11 comparisons across the defocus curve reduces the risk of type I error but increases the risk of type II error. Consequently, some numerically meaningful BSR_VA_ differences beyond the central defocus range may not have reached statistical significance despite representing a real effect worth confirming in a larger cohort. Finally, the binocular advantages reported here reflect group-level mean differences; individual participants showed variability in the magnitude, and in some cases the direction, of binocular summation (Table 2 and Table 4), and this study does not establish an individual-level prediction rule for who will benefit most from binocular correction strategies.

## 5. Conclusions

Binocularity provides a consistent and clinically meaningful enhancement of visual performance in healthy presbyopic subjects across the full range of distances evaluated by defocus curves. The binocular advantage in visual acuity was most consistent within the −0.50 D to +1.00 D defocus range in the planned pairwise contrasts and more variable at higher levels of negative defocus (near distances), where intersubject differences become more pronounced. However, the repeated-measures interaction between condition and defocus was not statistically significant, indicating no confirmed defocus-dependent modulation of this advantage. Binocularity was associated with a significant expansion of the optimal depth of focus (0.1 logMAR threshold) and a non-significant trend toward expansion of functional depth of focus (0.2 logMAR threshold), both displacing cut-off points toward more negative defocus values. Binocular summation of contrast sensitivity is large, robust and distance-independent, and remains functionally distinct from the summation pattern observed for visual acuity, in line with the different neural mechanisms underlying these two visual functions. These results contribute normative binocular defocus curve data for the 45–65-year presbyopic population and support the systematic assessment of binocular function beyond monocular dominant-eye measurements in clinical and research contexts involving this age group. These findings support the incorporation of binocular functional assessment into the personalized evaluation of presbyopic patients.

## Figures and Tables

**Table 1:** Descriptive statistics for the study sample. Monocular data for the distance dominant eye.

Parameter	Mean ± SD	Range [Min/Max]
Age (years)	53.73 ± 5.71	[45/64]
Sex (F/M)	16 (53.3%)/14 (46.7%)	-
SE (D)	−0.64 ± 2.06	[−6.00/+4.25]
Astigmatism (D)	−0.66 ± 0.67	[−3.00/0.00]
mCDVA at 4 m (logMAR)	−0.14 ± 0.07	[−0.28/0]
bCDVA at 4 m (logMAR)	−0.18 ± 0.06	[−0.28/−0.02]

F: Female; M: Male; SE: spherical equivalent; D: diopter; mCDVA: monocular corrected distance VA; bCDVA: binocular corrected-distance VA; SD: standard deviation.

**Table 2:** CS parameters and BSR at 1 m and 40 cm.

Parameter	1 m	40 cm	*p*-Value (40 cm vs. 1 m)
Monocular CS (logCS)	1.75 ± 0.17 [1.35/1.95]	1.69 ± 0.14 [1.35/1.95]	-
Binocular CS (logCS)	1.94 ± 0.06 [1.65/2.10]	1.91 ± 0.09 [1.65/2.10]	-
*p*-value (mono vs. bino)	*p* < 0.001	*p* < 0.001	-
Cohen’s d	1.11	1.97	
BSR_CS_	1.68 ± 0.72 [1.00/3.98]	1.69 ± 0.44 [1.00–2.82]	0.915

CS: contrast sensitivity; BSR: binocular summation ratio; m: meter. Statistical significance for monocular vs. binocular CS comparisons was assessed using a Bonferroni-corrected threshold of α = 0.025 (family of two tests); the between-distance BSR comparison was evaluated as a single test at α = 0.05.

**Table 3:** Monocular and binocular VA (mean ± SD, logMAR) at each defocus level, with paired *t*-test and Wilcoxon signed-rank results, Cohen’s d, and 95% confidence intervals for the mean difference. Asterisks indicate statistically significant paired monocular–binocular differences based on the Bonferroni-corrected significance threshold.

Defocus (D)	Monocular VA Mean ± SD	Binocular VA Mean ± SD	*p*-Value	Wilcoxon *p*-Value	Cohen’s d	95% CI of the Difference
−3.50	0.66 ± 0.21	0.63 ± 0.17	0.490	0.347	0.13	−0.045 to 0.092
−3.00	0.55 ± 0.20	0.50 ± 0.18	0.063	0.076	0.35	−0.003 to 0.095
−2.50	0.42 ± 0.20	0.38 ± 0.19	0.047	0.039	0.38	0.001 to 0.085
−2.00	0.32 ± 0.18	0.26 ± 0.19	0.013	0.007	0.48	0.013 to 0.097
−1.50	0.20 ± 0.17	0.14 ± 0.17	0.005	0.006	0.56	0.022 to 0.108
−1.00	0.07 ± 0.12	0.02 ± 0.12	0.005	0.002	0.56	0.017 to 0.083
−0.50	−0.06 ± 0.08	−0.10 ± 0.07	<0.001 *	0.001 *	0.70	0.018 to 0.059
0.00	−0.14 ± 0.07	−0.18 ± 0.06	<0.001 *	<0.001 *	1.16	0.026 to 0.051
+0.50	−0.03 ± 0.09	−0.08 ± 0.09	<0.001 *	<0.001 *	0.72	0.026 to 0.082
+1.00	0.16 ± 0.13	0.09 ± 0.14	0.001 *	0.002 *	0.65	0.029 to 0.106
+1.50	0.34 ± 0.12	0.30 ± 0.15	0.026	0.024	0.43	0.006 to 0.084

VA: visual acuity; SD: standard deviation; D: diopter. Monocular and binocular VA were compared using paired-samples Student’s *t*-tests and Wilcoxon signed-rank tests, given non-normal paired differences at several defocus levels. Cohen’s d was computed as the mean paired difference (monocular–binocular) divided by the SD of the paired differences; a positive value indicates better (lower) binocular logMAR. The 95% CI corresponds to the mean paired difference. *p*-values shown are raw (unadjusted) values compared against a Bonferroni-corrected significance threshold of α = 0.0045 for 11 comparisons, not adjusted *p*-values. *: Significant at this threshold.

**Table 4:** BSR values (mean ± SD) and percentage binocular improvement at each defocus level. Percentage improvement = (BSR − 1) × 100.

Defocus (D)	BSR_VA_ (Mean ± SD)	95% CI	Improvement (%)
−3.50	1.14 ± 0.45	0.97–1.31	14%
−3.00	1.16 ± 0.36	1.03–1.30	16%
−2.50	1.14 ± 0.32	1.02–1.26	14%
−2.00	1.17 ± 0.31	1.06–1.29	17%
−1.50	1.21 ± 0.38	1.06–1.35	21%
−1.00	1.15 ± 0.27	1.05–1.25	15%
−0.50	1.10 ± 0.16	1.04–1.16	10%
0.00	1.10 ± 0.09	1.06–1.13	10%
+0.50	1.15 ± 0.23	1.06–1.24	15%
+1.00	1.20 ± 0.30	1.09–1.31	20%
+1.50	1.14 ± 0.28	1.04–1.24	14%

VA: Visual acuity; D: diopter; BSR: binocular summation ratio; SD: standard deviation; CI: confidence interval. The width of these intervals, several of which approach or include values close to 1 at the peripheral defocus levels, corroborates the decision to base statistical significance on the direct monocular-versus-binocular VA comparison (Table 3) rather than on testing BSR against unity, since BSR is a ratio-derived, more variable metric.

## Data Availability

The datasets generated and analyzed during the current study are openly available in GitHub at https://github.com/NuriaGarzonUCM/Bino-Defocus-Curve.git (Accessed on 22 July 2026).
